# Preoperative Temporal Elevation of Cytokeratin 19 Fragment in Thymoma With Extensive Necrosis: A Case Report

**DOI:** 10.1111/1759-7714.70409

**Published:** 2026-10-31

**Authors:** Yuki Nishioka, Tappei Shomoto, Sanae Kuroda, Megumi Nishikubo, Nahoko Shimizu, Keiichi Morita, Kazuyoshi Kajimoto, Watatu Nishio

**Affiliations:** ^1^ Division of Chest Surgery Hyogo Cancer Center Akashi Hyogo Japan; ^2^ Division of Pathology Hyogo Cancer Center Akashi Hyogo Japan

**Keywords:** case report, cytokeratin fragment, necrosis, thymoma

## Abstract

Thymoma with necrosis is rare. Due to the presence of necrosis, a preoperative diagnosis is challenging to make. Cytokeratin 19 fragment (CYFRA) is reportedly low in thymoma. While CYFRA elevation has been detected when in combination with necrosis, the preoperative transition has rarely been described. The patient was a 73‐year‐old man who presented with chest pain. An initial computed tomography (CT) scan showed a mediastinal tumor accompanied by pleural effusion. In a serological examination, in addition to elevated white blood cell count (WBC) and C‐reactive protein (CRP), CYFRA was elevated (61.3 ng/mL). A second CT examination revealed the disappearance of pleural effusion without a decrease in mass size. His laboratory markers decreased without treatment. Preoperatively, his CYFRA level was 38.7 ng/mL. Thymectomy was performed via median sternotomy, with partial resection of pericardium. A pathological examination revealed thymoma, suggestive of type B, with Masaoka stage II, accompanied by extensive necrosis. There were no signs of recurrence at 5 months postoperatively. A temporal increase in CYFRA before surgery may imply necrosis of thymic tumor cells. Thymoma with necrosis should be considered as a differential diagnosis.

## Graphical abstract

In a mediastinal tumor accompanied by inflammation and a low‐density area, cytokeratin 19 fragment (CYFRA) was elevated, but it decreased preoperatively. The pathological diagnosis was thymoma with extensive necrosis (possibly Type B). A temporal CYFRA elevation may indicate tumor necrosis. Thymoma with necrosis should be considered in the differential diagnosis.

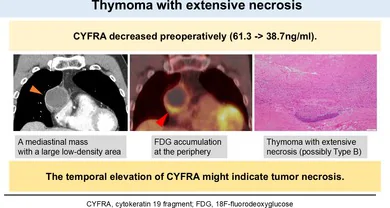

## 1 Introduction

Thymoma is the most prevalent thymic malignancy, accounting for 20% of mediastinal malignancies [1]. Necrosis or hemorrhage is detected in 5% of thymomas [2]. In thymoma with extensive necrosis, inflammation‐related symptoms (e.g., chest pain or fever) have been accompanied by elevation of inflammatory markers (e.g., white blood cell [WBC] count and C‐reactive protein [CRP] level). These clinical characteristics mimic those of infectious thymic cysts or ruptured teratomas. A preoperative diagnosis is challenging to make due to the presence of necrosis. Cytokeratin 19 fragment (CYFRA) is an epithelial tumor marker that can be elevated in thymic epithelial malignancies. In thymoma, the levels are typically low due to its indolent growth [3]. Although elevation has been reported in thymomas with necrosis, preoperative fluctuations have rarely been described. We present a case of thymoma with temporal marker elevation prior to surgery. We also discuss the possible mechanisms underlying this elevation and the relevance of CYFRA in the differential diagnosis.

## 2 Case Report

The patient was a 73‐year‐old man who presented with chest pain. Laboratory data showed WBC and CRP elevation. Plain CT showed a mediastinal mass (54 × 45 mm) and right pleural effusion. At referral to our hospital (a tertiary center, Hyogo, Japan) 1 week later, these inflammatory markers and the anti‐acetylcholine receptor (AChR) antibody level were within the normal range; however, CYFRA was elevated (61.3 ng/mL). Contrast‐enhanced CT showed a mass that included a large low‐density area in the center, which had not shrunk in size (Figure 1A). The pleural effusion had disappeared. 18F‐fluorodeoxyglucose (FDG) positron emission tomography (PET) showed the uptake of FDG with a maximum standardized uptake value (SUVmax) of 4.9 (Figure 1B). One month after the onset of the symptoms, the patient's CYFRA decreased (38.7 ng/mL). A resectable thymic epithelial tumor was suspected, and surgical resection for both diagnostic and treatment reasons was proposed. The patient accepted the need for surgery and consented to the treatment plan. Thymectomy was performed through median sternotomy. The tumor was slightly adhesive to the right lung but easily dissected. However, the tumor firmly adhered to the pericardium, requiring an en bloc resection of the pericardium followed by reconstruction with an expanded polytetrafluoroethylene (ePTFE) membrane (0.1 mm‐thickness; W.L. Gore & Associates Inc., Japan). Macroscopically, the tumor measured 50 mm and contained a yellow‐white area (Figure 2A). The pathological diagnosis was thymoma with extensive necrosis (Figure 2B). In immunohistochemistry, viable cells were positive for terminal deoxynucleotidyl transferase (TdT) and β5t (Figure 2C,D). Its subtype was indeterminate due to scarce viable cells; however, the positivity for β5t suggested its subtype was type B. No invasion to the adjacent pericardium was observed. The CYFRA level returned within the normal range at 1.5 months postoperatively. A chronological timeline of the clinical course and serological markers is shown in Figure 3. The postoperative course was uneventful with no evidence of recurrence at 5 months postoperatively. At the follow‐up visit, the patient stated that he was grateful for the decision to undergo surgical resection since it had led to the final diagnosis and successful treatment.

**FIGURE 1:**
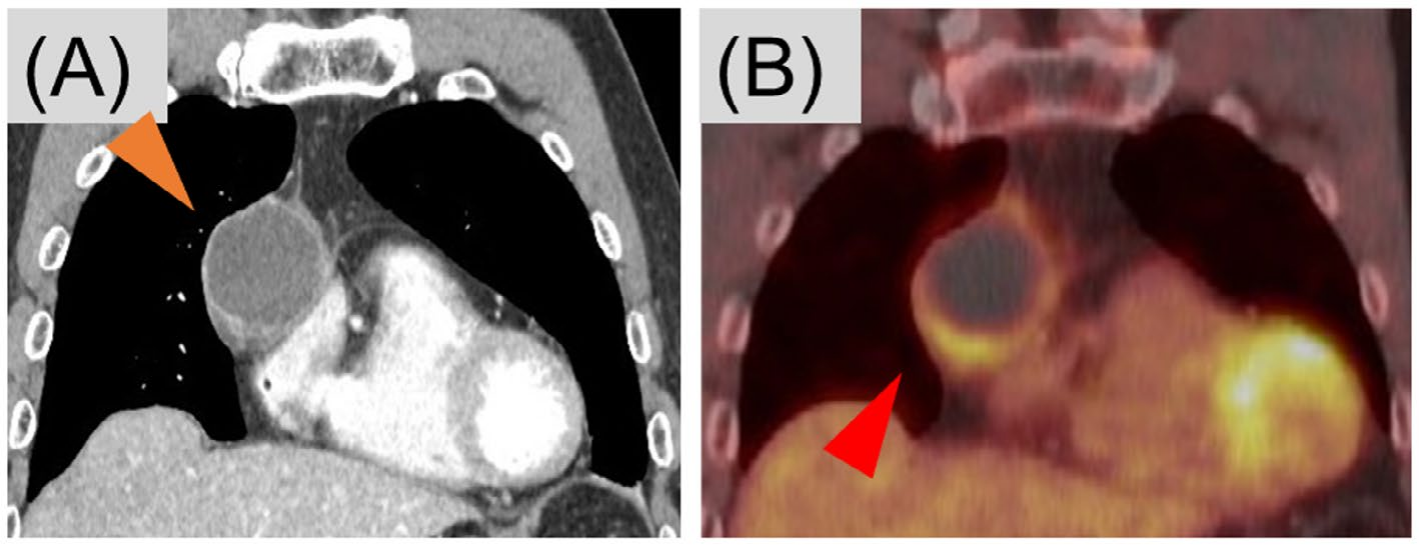
Radiological findings. (A) Enhanced computed tomography (CT) showed a cystic mass (orange arrowhead) measuring 54 × 45 mm in the anterior mediastinum, with peripheral enhancement. (B) ^18^F‐fluorodeoxyglucose (FDG) positron emission tomography (PET) showed the uptake of FDG with a maximum standardized uptake value (SUVmax) of 4.9 (red arrowhead).

**FIGURE 2:**
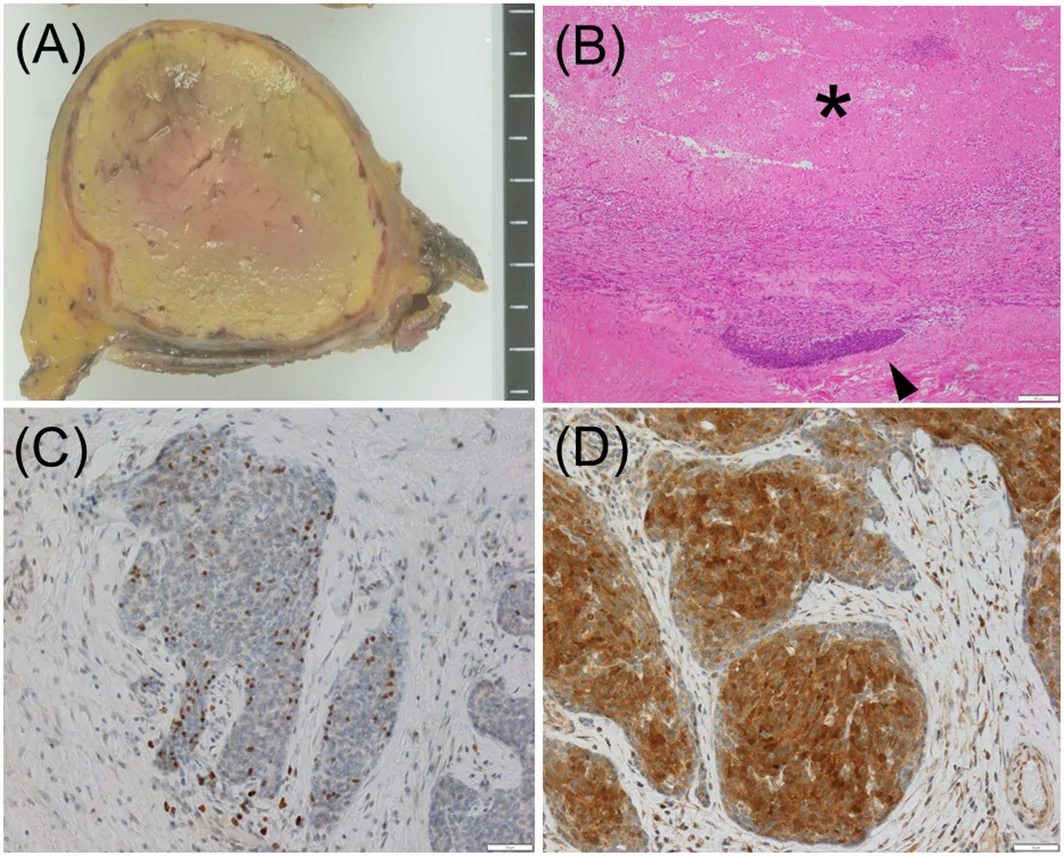
Pathological findings. (A) The cut surface showed the tumor was encapsulated with a white inner area. (B) Hematoxylin and Eosin staining (low power view) showed the necrotic area (*) and few viable cells (arrowhead). Immunohistochemistry demonstrated positivity for (C) terminal deoxynucleotidyl transferase (TdT) and (D) β5t.

**FIGURE 3:**
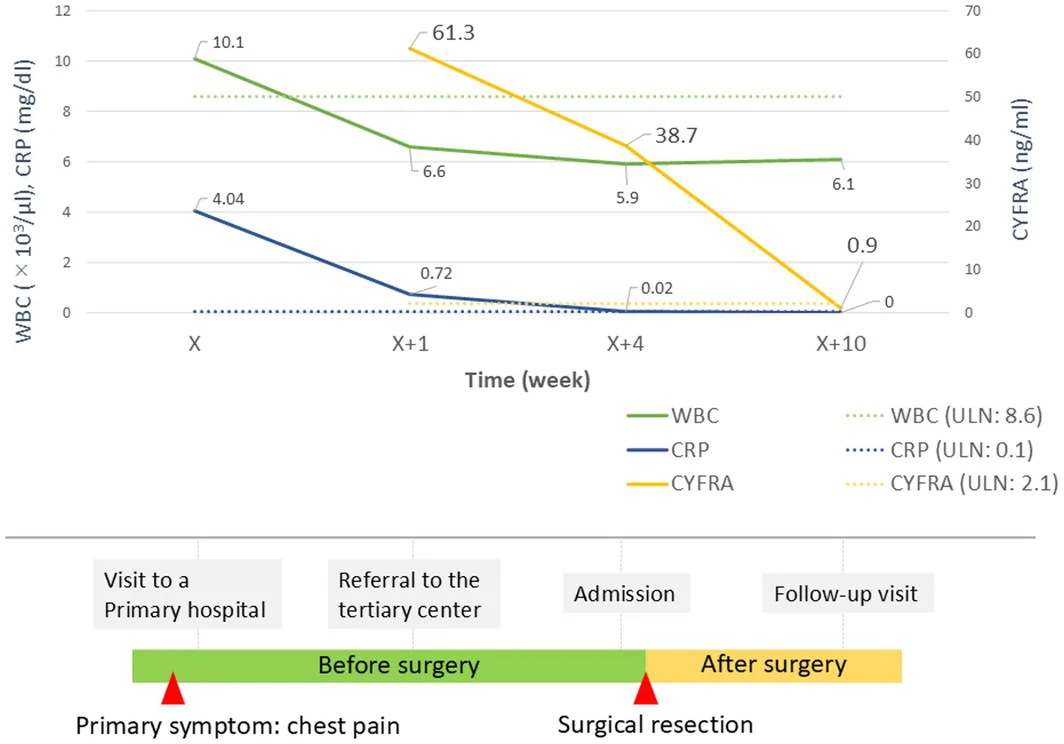
Chronological timeline of the clinical course and serological markers. When the patient initially presented to a primary hospital, the WBC and CRP levels were elevated. At the referral to the tertiary center, these levels had decreased, but the CYFRA level was elevated. Preoperatively, the CYFRA level decreased without any medication. The postoperative course was uneventful, and the transient postoperative elevation of the WBC and CRP levels, induced by surgery, was omitted. Solid lines show the measured values of each marker. Dotted lines of the corresponding color indicate the upper limit of normal. CRP, C‐reactive protein; CYFRA, Cytokeratin 19 fragment; ULN, upper limit of normal; WBC, white blood cell.

## 3 Discussion

Table 1 summarizes the reports on cases with preoperative CYFRA elevation, including this case [4, 5, 6, 7, 8, 9, 10, 11]. The present case exhibited a similar clinical course, with acute symptoms, inflammatory marker elevation, and temporal pleural effusion.

**TABLE 1:** Reported cases of thymoma with necrosis accompanied by preoperatively elevated CYFRA.

Author, year	Age/sex	Symptoms		Serological findings			Radiological findings			Pre‐operative biopsy	Pathological findings			Post‐operative course	
		Chest pain	Fever	Elevation of WBC or CRP	Anti Ach‐R antibody	CYFRA (ng/ml)	Tumor size (mm)	SR	PE		Necrosis	Masaoka stage	WHO type	Adjuvant treatment	Prognosis: no recurrence
Ishibashi, 2003 [4]	44/F	(+)	(−)	(+)	—	4.1	70	(+)	(+)	Not‐diagnostic	(+)	I	B2	(−)	3 years
Yutaka, 2009 [5]	47/M	(+)	—	(−)	—	40	80	(+)	(+)	Thymic carcinoma	(+)	III	B3	RT	1 year
Himuro, 2014 [6]	73/F	—	—	(+)	—	29	65	(+)	—	—	(+)	I	B2	(−)	5 months
Shiiya, 2015 [7]	53/M	(+)	(+)	(+)	(−)	19	57	(+)	(+)	—	(+)	II	AB	(−)	—
Furuya, 2015 [8]	30/M	(+)	(+)	(+)	(−)	13 to 0.8	110	(+)	(+)	Necrotic	(+)	II	B2	(−)	1 year
Tsunooka, 2015 [9]	65/M	—	(−)	(+)	—	4.4 to WNL	71	(+)	(+)	Necrotic	(+)	II	B3	RT	3 years
Hinokuma, 2020 [10]	36/M	(+)	(+)	(+)	(+)	59	60	(+)	(+)	Atypical cells	(+)	II	B2	(−)	1 year
Tokunaga, 2025 [11]	30/M	(+)	(+)	(+)	(+)	4.2	88	(+)	(−)	—	(+)	II	B2 + B3	(−)	11 months
Our case	73/M	(+)	(−)	(+)	(−)	61 to 39	54	(−)	(+)	—	(+)	IIa	B	(−)	5 months

Abbreviations: (−), absent; (+), present; —, not available; Ach‐R, acetylcholine‐receptor; CRP, C‐reactive protein; CYFRA, Cytokeratin 19 fragment (CYFRA); PE, pleural effusion; RT, radiation therapy; SR, spontaneous regression; WBC, white blood cell; WHO, world health organization; WNL, within the normal range.

The median CYFRA level in thymoma is reported to be 1.2 ng/mL [3]. For thymic malignancies, a cut‐off value of 2.7 ng/mL was proposed to distinguish thymoma from thymic carcinoma. In addition to the CYFRA level, the maximum standard uptake volume (SUVmax) on PET may provide a diagnostic clue, with values > 8 indicating the possibility of thymic carcinoma [12]. In this case, the serological and radiological findings were insufficient to establish a definitive diagnosis.

We performed upfront surgery base on the following three reasons. First, from an epidemiologic view, germ cell tumors were often diagnosed at a younger age than in this case. Second, a preoperative needle biopsy has been presumed to yield a non‐diagnostic result due to the presence of necrosis and the elevated CYFRA level implied the possibility of a thymic epithelial tumor. Third, the tumor had been assessed as completely resectable.

In a previous case of necrotic thymoma with pleural effusion, CYFRA was elevated in both serum and pleural effusion, with no evidence of pleural dissemination [8]. The serum level decreased to the normal range when the tumor size decreased, along with the disappearance of pleural effusion. The elevation was attributed to tumor necrosis. In another study, spontaneous regression was detected and CYFRA was elevated preoperatively, but preoperative transition was not described [11]. In addition, on radiological imaging, spontaneous shrinkage is presumed to be a pathological consequence of necrosis or hemorrhage [9]. In our case, a consequent decrease in CYFRA without a decrease in tumor size, accompanied by necrosis, was detected. Considering the similarity in the pathological findings, as a possible explanation for the discordances between our case and previous reports, we presume that the relevant changes occurred at different time points: pathological necrosis first, followed by serological CYFRA elevation, and ultimately radiological tumor shrinkage. This hypothesis should be warranted in additional cases. In cases with radiological findings suggesting necrosis, preoperative pathological confirmation is difficult. This case highlighted that the preoperative fluctuation of CYFRA served as a clue for the clinical diagnosis of thymic epithelial tumor: the remarkable elevation had not been caused by thymic carcinoma.

Several mechanisms of necrosis have been proposed. Insufficient vascular supply due to rapid tumor growth has been considered to induce ischemic changes [8, 13, 14]. Another mechanism is thrombus formation, which can trigger ischemic infarction and necrosis [15, 16]. In our cases, pathological evaluation revealed widespread coagulative necrosis in the absence of thrombus or hemorrhagic changes, with only scant viable cells remaining at the periphery. The presence of coagulative necrosis may imply the absence of a sudden occlusive event; however, the underlying mechanism remains unclear.

In conclusion, in thymoma, the temporal CYFRA elevation may indicate tumor necrosis. In a mediastinal tumor accompanied by inflammation or radiological findings suspicious of necrosis, such as a low‐density area, CYFRA may provide clues for the differential diagnosis.

## Author Contributions

**Yuki Nishioka:** conceptualization, writing – original draft, writing – review and editing. **Tappei Shomoto:** writing – review and editing. **Sanae Kuroda:** writing – review and editing. **Megumi Nishikubo:** writing – review and editing. **Nahoko Shimizu:** writing – review and editing. **Keiichi Morita:** writing – review and editing. **Kazuyoshi Kajimoto:** supervision. **Watatu Nishio:** supervision.

## Funding

The authors have nothing to report.

## Ethics Statement

This case report was approved by the Institutional Review Board of Hyogo Cancer Hospital.

## Consent

Written informed consent was obtained from the patient.

## Conflicts of Interest

The authors declare no conflicts of interest.

## Data Availability

The data that support the findings of this study are available from the corresponding author upon reasonable request.
